# Photodynamic Therapy

**DOI:** 10.3390/biomedicines10112701

**Published:** 2022-10-26

**Authors:** Kyungsu Kang, Stefano Bacci

**Affiliations:** 1 Natural Product Informatics Research Center, Gangneung Institute of Natural Products, Korea Institute of Science and Technology, Gangneung 25451, Gangwon-do, Korea; 2 Division of Bio-Medical Science & Technology, KIST School, University of Science and Technology (UST), Gangneung 25451, Gangwon-do, Korea; 3 Research Unit of Histology and Embriology, Department of Biology, University of Florence, Viale Pieraccini 6, 50139 Florence, Italy

In 1903, Von Tappeiner and Jesionek [1] showed the efficacy of light treatment together with a photosensitizer and oxygen, which is so-called “photodynamic action”. Medically, photodynamic therapy (PDT) application is now extensively used, and PDT has been exploited to treat various oncological and non-oncological human diseases.

This editorial provides an up-to-date brief review of “Photodynamic therapy” appearing in biomedicine and some recent studies related to this therapy. We summarize the various contributions highlighting new analytic approaches and updates related to photodynamic therapy. The editorial is dedicated to concise updates on general or specific arguments regarding the utility of photodynamic therapy.

**Review of General Arguments**

**About tumors**: since the rate of survival for subjects with tumors like glioblastoma multiforme (GBM) greater risk malignancy is below a year, clinical outcomes of patients with GBM who received PDT may be improved through the use of nanomedicine. The review by Kim and Lee summarizes the utility of clinical PDT applications of nanomedicine for the management of GBM (for more information, see [2]). Matsuoka et al., reviewed the role of PDT for the management of early stage malignant lung tumors. Recently, a near-infrared photoimmunotherapy (NIR-PIT) has been developed for the treatment of recurrent head and neck malignancies due to its specificity and efficacy. In this review, NIR-PIT is introduced, and its potential therapeutic utilities for thoracic cancers are elaborated (for more details, see [3]).

**About SARS-CoV-2**: Balhaddad et al., discuss the current efforts and limitations on utilizing biophotonic approaches to prevent the transmission of SARS-CoV-2 in dental care and provide relevant information regarding the intricacies and complexities of infection control in dental care (for more details, see [4]).

**On chronic wounds**: since wound healing process involves a complex interplay and organization of different cells and biomolecules and any modification with these extremely organized events may cause prolonged or excessive healing, Grandi et al., elucidated the cellular mechanisms described, upon therapy with 5-aminovuleinic acid (ALA)-PDT, in chronic wounds, that may be linked with social isolation and high costs (for more details, see [5]).

**Research Articles of Specific Topics**

**About the studies on transplantation**: Lin et al., explore the possibility of far-infrared (FIR) in preventing orthotopic allograft transplantation (OAT) using an aorta graft from PVG/Seac to ACI/NKyo rats, and human endothelial progenitor cells (EPC). The authors reported that FIR treatment decreased vasculopathy in OAT-recipient ACI/NKyo rats, as well as the immune responses mediated by the spleen and the release of serum inflammatory markers. Higher mobilization and circulating EPC levels related to vessel repair in OAT-recipient ACI/NKyo rats were seen. In vitro studies showed that this process may be related to the blockade of the Smad2-Slug signaling axis of endothelial mesenchymal transition. The authors proposed that FIR therapy can be considered a potential strategy to mitigate chronic rejection-induced vasculopathy (for more details, see [6]).

**About the studies on periodontal treatments**: Solarte et al., evaluated the antimicrobial effect and cytotoxicity of PDT with indocyanine green with visible light and water-filtered infrared A in patients with chronic periodontitis. Almost all bacterial pathogens were eliminated as well as significant differences were found in the subgingival biofilms. The authors propose this photodynamic therapy as an adjuvant to periodontal treatments (see [7] for additional details).Tisler et al., assessed the effect of PDT to improve the bond strength of full ceramic restorations. Their investigation demonstrates that the bacterial number was decreased from colonized marked tooth exteriors, manifesting with a rise in the bond strength following the PDT treatment. The authors indicate that PDT treatment before the final adhesive cementation of ceramic restorations may be an optimistic strategy compared to conventional practice (for more information, see [8]).

**About the studies on tumors**: Cacaccio et al., studied the efficacy of PDT with a non-radioactive sensitizer (PS) in tumors derived from lung cancer patients in mice models. The in vitro and in vivo efficacy was also evaluated in combination with doxorubicin, and long-term tumor response was significantly increased. The authors propose that the iodinated PS is efficient for the treatment of lung tumors (for additional information, see [9]). Lamy et al., studied if the use of hexaminolevulinate and blue light cystoscopy in an orthotopic rat model of bladder cancer may have therapeutic efficacy by modulation of a tumor-specific immune response and measured if its delivery in combination with a checkpoint inhibitor may enhance any effects observed. Positive anti-tumor effect was related to the timing of the process with a localization of CD3+ and CD8+ cells at long term: the effect was increased when delivered in combination with intravesical anti-PD-L1 (for more information, see [10]). Klimenko et al., describe the activity of (3S,4S)-14-Ethyl-9-(hydroxymethyl)−4,8,13,18-tetramethyl-20-oxo-3-phorbinepropanoic acid (ETPA) as a crucial metabolite of the North Pacific brittle stars *Ophiura sarsii* in a mouse model of glioblastoma. Intravenous ETPA administered in addition with a targeted red laser irradiation induced strong necrotic ablation of glioblastoma. The authors propose ETPA as a natural product-based photodynamic drug (for more information, see [11]). Pevna et al., measured the efficacy of hypericin-mediated PDT in U87 MG cells human GBM cells subjected to the treatment with rotenone that influence their metabolic activity. This treatment stimulates autophagy and increases the anticancer efficacy and leads to apoptosis. This seems to decrease the damage in surrounding normal tissues when hypericin-PDT is utilized for in vivo tumor treatments (For more information, see [12]). Vasilev et al., demonstrate that tetramethylrhodamine methyl ester (TMRM), which is a fluorescent dye to evaluate mitochondrial potential, may be utilized as a photosensitizer to select GBM cells. The results show that PDT with TMRM and low-intensity green light stimulated mitochondrial damage and led to GBM cell death, but not cultured rat astrocytes. The authors propose that TMRM as a mitochondrially targeted photosensitizer may be considered for preclinical or clinical studies (for more information, see [13]). Chiang et al., attempted to make the processes associated with PDT-mediated chloride intracellular channel (CLIC4) inhibition in human melanoma A375 cells and in human breast cancer MDA-MB-231 cells clear. The findings show the increase of the release and enzymatic effects of DNA methyltransferase 1 (DNMT1), the hypermethylation in the *CLIC4* promoter region and the involvement of P53 in the higher DNMT1 release in PDT-treated cells. The authors propose that CLIC4 suppression induced by PDT is modulated by DNMT1-mediated hypermethylation and revolves around p53, which suggests a coordinated process for regulating CLIC4 release in tumorigenesis (for more information, see [14]). Magalhaes et al., assessed the heavy-atom effect (HEA) as a mechanism for anticancer activity to coelenterazine derivatives, a chemiluminescent molecule widespread in marine organisms. The study findings suggest the use of HEA facilitates these molecules to manifest readily available triplet states in a chemiluminescent reaction stimulated by a tumor marker, but the potency of the anticancer activity is determined by the tumor type. The authors propose that the utilization of the HEA to marine coelenterazine may be an optimistic strategy for the development of new tumor-selective sensitizers for light-free PDT (for more information, see [15]).

**About the studies on the efficacy of photosensitizers**: Desgranges et al., studied a series of amphiphilic protoporphyrin derivatives since protoporphyrin IX (PpIX), is limited in clinical PDT by relative insolubility in watery and the possibility to self-aggregate. The findings demonstrate the therapeutic potency of these new PpIX because some of them demonstrated a higher photodynamic activity compared to the parent PpIX (for more information, see [16]). Mantareva et al., present the efficacy of a new sensitizer with peripheral positions of methylpiridoxy substitution groups (pPdPc and ZnPcMe) on Gram-negative bacteria *Aeromonas hydrophila*, antibiotic-resistant and sensitive strains. The photoinactivation demonstrated a complete activity with 8 µM pPdPc for antibiotic-sensitive strain and with 5 µM ZnPcMe for both antibiotic-resistant and sensitive strains. These results suggest that the uptakes and photoinactivation efficacy of the applied phthalocyanines are not related to the drug sensitivity of both strains (for more information, see [17]). In a study by Ramachandran et al., TiO_2_ NPs and TiO_2_ conjugated with N-GQDs/TiO_2_ NCs were synthetized via microwave-assisted synthesis and two-pot hydrothermal method, respectively. Upon the photo-activation with near-infrared (NIR) light, the nanocomposites elaborated reactive oxygen species (ROS), which caused more significant mitochondria-associated apoptotic cell death in human breast cancer MDA-MB-231 cells than in human foreskin fibroblast HS27 cells. The authors propose that titanium dioxide-based nanocomposite upon photoactivation is a possible photosensitizer for PDT against human breast cancer care (for more information, see [18]). Fang et al., produced a styrene maleic acid copolymer (SMA) micelle encapsulating temoporfin (mTHPC), which is medically a PDT drug. SMA@mTHPC, showed a pH-dependent release profile, and higher expression took place at acidic pH, indicating that marked expression of free mTHPC may take place in the weak acidic pH setting of cancers and more so during internalization into cancer cells. In vitro cytotoxicity assay indicated a smaller activity of SMA@mTHPC compared to free mTHPC; but severe side effects were observed during free mTHPC treatment to the contrary of SMA@mTHPC. The better safety profile of SMA@mTHPC was mainly because of its micelle formation and the increased permeability and retention effect-based tumor accumulation, and the tumor environment-responsive release properties. These observations indicated that SMA@mTHPC may be PDT drugs for targeted tumor treatment with a lower side effect (for more details, see [19]). Polat and Kang, reviewed natural photosensitizers and synthetic derivatives photosensitizers for antimicrobial photodynamic therapy (APDT) to control various pathogenic organisms. Regards to natural photosensitizers, many single compounds, as well as many plant extracts have been used for photosensitizers for APDT. Preclinical experimental models using a model nematode, *Caenorhabditis elegans*, wax moth, in addition to rodent model are used to evaluate the efficacy and side effects of new APDT. Various emerging technologies such as cell surface and protein engineering, photosensitizer uptake strategies, nano-delivery systems, and computational simulation are introduced in this review (for more information, see [20]). Alam et al., reported natural photosensitizers prepared from the medicinal plant *Tripterygium wilfordii* for antimicrobial photodynamic purposes. Ethanol extract (TWE) and a photosensitizer-enriched fraction contain six pheophorbide derivatives as active compounds. Cotreatment of red light (660 nm, 120 W/m^2^) and natural photosensitizers (TWE) potently killed pathogenic bacteria and fungi, especially various skin pathogens in vitro. Their in vivo APDT efficacies and adverse effects were assessed using the model nematode *C. elegans* infected with *Staphylococcus aureus* and *Streptococcus pyogenes*, which are representative skin pathogens (for more information, see [21]).

**About the studies on the cellular mechanisms evoked by PDT**: Espeland et al., studied the effects of ALA-PDT on cytokines and exosomes of human peripheral blood mononuclear cells. The therapy appeared to lower all pro-inflammatory cytokines, indicating that the PDT may lead to a strong anti-inflammatory effect. In addition, the therapy lowered the levels of different types of exosomes, in particular the HLA-DRDPDQ exosome, which is very crucial in the rejection process of organ transplantation and autoimmune diseases. Their study suggests future therapeutic strategies of ALA-PDT for modulation of immune systems (for more details about this article see [22]).

**Discussion**

This research topic gives an opportunity for the meeting of experts on photodynamic therapy. The reviews proposed are clear in their content and the research articles cover new approaches and therapeutic targets that will certainly be deepened in future. In conclusion, from these studies, it emerges that through this therapy there is more hope in the clinical field for the eradication of diseases that have always pursued humanity.
